# CaMKII is involved in subcellular Ca^2+^ redistribution-induced endoplasmic reticulum stress leading to apoptosis in primary cultures of rat proximal tubular cells exposed to lead

**DOI:** 10.18632/oncotarget.20035

**Published:** 2017-08-08

**Authors:** Min-Ge Wang, Wen-Hui Li, Xin-Yu Wang, Du-Bao Yang, Zhen-Yong Wang, Lin Wang

**Affiliations:** ^1^ College of Animal Science and Veterinary Medicine, Shandong Agricultural University, Tai’an City, Shandong Province, 271018, China; ^2^ Shandong Provincial Key Laboratory of Animal Biotechnology and Disease Control and Prevention, Shandong Agricultural University, Tai’an City, Shandong Province, 271018, China; ^3^ Shandong Provincial Engineering Technology Research Center of Animal Disease Control and Prevention, Shandong Agricultural University, Tai’an City, Shandong Province, 271018, China

**Keywords:** lead, calcium, proximal tubular cells, CaMKII, endoplasmic reticulum stress

## Abstract

Lead (Pb) is a known nephrotoxic element. Recently we have proved that subcellular Ca^2+^ redistribution is involved in Pb-induced apoptosis in primary cultures of rat proximal tubular (rPT) cells, but the underlying mechanism remains to be elucidated. Firstly, data showed that Pb triggers endoplasmic reticulum (ER) stress response in rPT cells, as evidenced by the elevations of ER stress markers. Moreover, pharmacological modulation of Ca^2+^ mobilization in ER and cytoplasm with three chemicals (2-APB or TG or BAPTA-AM) can effectively increase or decrease the protein expression of ER stress markers in Pb-exposed rPT cells, demonstrating that Pb-induced ER stress is Ca^2+^-dependent. We found that Pb stimulates phosphorylation of calcium/calmodulin-dependent protein kinase II (CaMKII) to activate its activity. Meanwhile, inhibition of CaMKII with KN93 or KN62 attenuated Pb-activated caspase-12 and CCAAT/enhancer-binding protein homologous protein (CHOP) in rPT cells, demonstrating that CaMKII activation promoted ER stress in rPT cells. Likewise, Pb-induced apoptosis can be effectively inhibited by CaMKII inhibitor KN93 or KN62. Furthermore, co-treatment with KN93 or KN62 significantly reversed Pb-induced ER Ca^2+^ release and concomitant intracellular Ca^2+^ overload in rPT cells. In summary, these results expound the mechanisms involving in ER stress, Ca^2+^ dyshomeostasis and activated CaMKII, which all contribute to Pb-induced apoptosis. CaMKII acts as a critical mediator of ER stress and associated apoptosis via regulating intracellular Ca^2+^ mobilization from ER to cytoplasm.

## INTRODUCTION

Lead (Pb) is a nonessential toxic heavy metal and one of the most widely used metals in industries. Globally, Pb exposure is ubiquitous and routes of its exposure to animals and human beings include inhalation of Pb-contaminated dust particles or aerosols, and ingestion of Pb-contaminated food or water [1]. The persistence of Pb in humans and its associated health risks are a matter of serious concern and a global issue. As a multi-organ toxicant, Pb exerts potent toxic effects on different tissues [2–7]. Kidney is one of the most sensitive targets organs for Pb toxicity, and the proximal tubule is the major site of Pb-induced renal injury [2, 4, 7]. Primary cultures possess more advantages compared to permanent cell lines in the toxicological research [8], thus primary rat proximal tubular (rPT) cells were established to elucidate low-level Pb-induced nephrotoxicity in this study.

Our research group have recently found that the apoptotic death triggered by subcellular Ca^2+^ redistribution played a chief role in low-dose (0–1.0 µM) Pb-induced nephrotoxicity in rPT cells [9]. Ca^2+^ ion is the most commonly employed signal transduction element in the process of apoptosis. Ca^2+^ is neither synthesized nor metabolized as other intracellular messengers, and its storage and mobilization is controlled by calcium channels, pumps and exchangers [10]. The endoplasmic reticulum (ER) is an essential intracellular organelle, responsible for intracellular Ca^2+^ homeostasis and protein folding and processing [11]. Dysfunction of Ca^2+^ homeostasis in the ER leads to the accumulation of unfolded proteins and activates the ER stress-induced apoptosis pathway [12]. Based on our previous results [9], we intend to investigate the correlation between Ca^2+^ dyshomeostasis and ER stress-mediated apoptosis in Pb-exposed rPT cells.

Moreover, Ca^2+^/calmodulin-dependent protein kinase II (CaMKII), a Ca^2+^-dependent protein kinase, is a key modulator of Ca^2+^ homeostasis [13]. CaMKII is characterized by its unique ability to decode and integrate oscillatory Ca^2+^ signals into specific outcomes through regulation of intracellular Ca^2+^ stores [14]. Sustained, excessive CaMKII activation is an upstream signaling event for promoting ER-Ca^2+^ release, while CaMKII inhibition markedly accelerates and amplifies ER Ca^2+^ stores [14]. Also, CaMKII is emerging as a critical mediator of ER stress and oxidative damage, while both pharmacological inhibition and genetic deletion of CaMKII have been shown to be protective against ER stress-induced apoptosis [15]. Our previous study has demonstrated that Pb disrupted the intracellular Ca^2+^ homeostasis in rPT cells [9], but the role of ER stress and CaMKII modulation in this process were not defined. This study will offer further evidences to clarify this question and investigate the role of CaMKII in Ca^2+^ mobilization, ER stress and apoptosis in Pb-exposed rPT cells.

## RESULTS

### Pb triggers ER stress response in rPT cells

**Figure 1:**
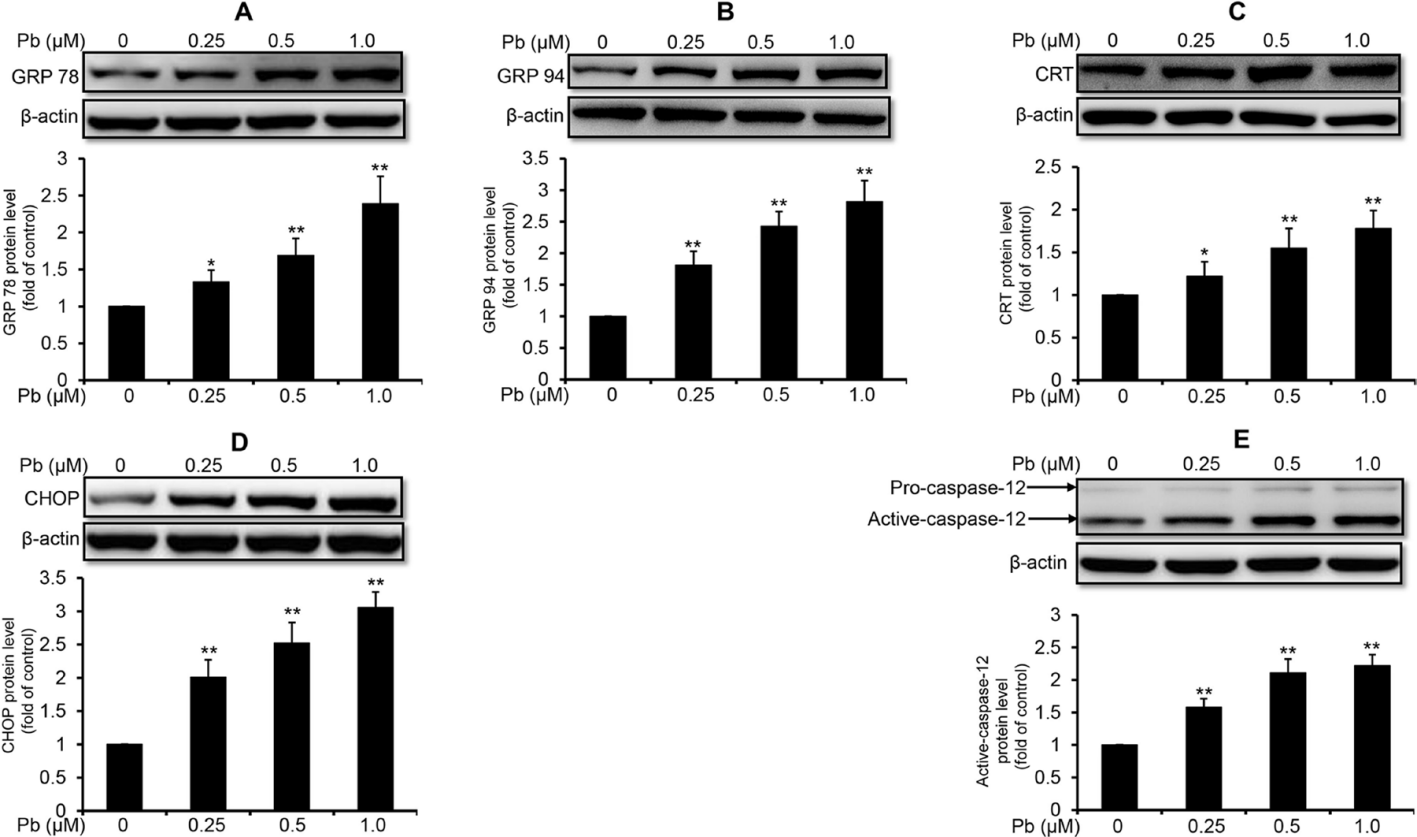
Pb elevated the protein levels of ER stress markers in rPT cells Cells were treated with Pb(NO_3_)_2_ (0.25, 0.5 and 1 µM) for 12 h, then collected to assess the protein levels of GRP78 (**A**), GRP94 (**B**), CRT (**C**), CHOP (**D**) and caspase-12 (**E**) using western-blot analysis. *Upper panel* representative western blot image; *lower panel* quantitative analysis (mean ± SEM, *n* = 4). ^*^P < 0.05, ^**^P < 0.01.

To investigate whether Pb induced ER stress in rPT cells, protein levels of several markers relevant to ER stress response were determined in this study. As shown in Figure 1, 12 h-Pb treatment caused a significant increase in the protein levels of glucose-regulated protein 78 (GRP78) (A), glucose-regulated protein 94 (GRP94) (B), calreticulin (CRT) (C), CHOP (D) and active caspase-12 (E) in rPT cells (*P* < 0.05), following by a dose-dependent manner, suggesting that Pb exposure induced the ER stress in rPT cells.

### Effects of 2-APB, TG and BAPTA-AM on the modulation of [Ca^2+^]_c_ and [Ca^2+^]_ER_

**Figure 2:**
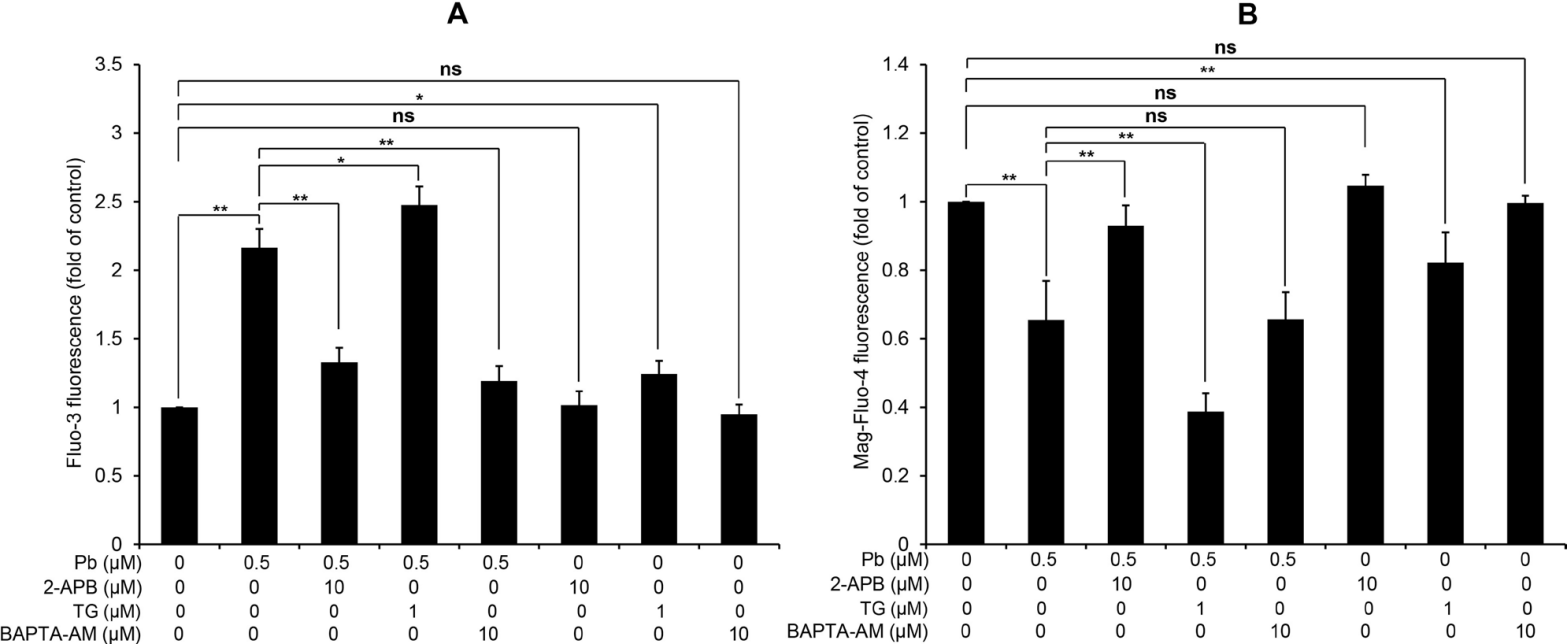
Modulation of [Ca^2+^]c and [Ca^2+^]ER by three regulators of Ca^2+^ signaling in rPT cells Cells were treated with 0.5 µM Pb for 12 h in the presence or absence of 10 µM 2-APB (co-incubation for the entire course), 1 µM TG (co-incubation for the entire course) and 10 µM BAPTA-AM (30 min pre-incubation before Pb treatment) to assess the changes of [Ca^2+^]_c_ and [Ca^2+^]_ER_, assessed by flow cytometry. Values of fluorescence intensity of Fluo-3 (**A**) and Mag-Fluo-4 (**B**) are quantified in a relative way to its respective control, whose value is set at one. Data represent mean ± SEM (*n* = 6). *ns* not significant, ^*^P < 0.05, ^**^P < 0.01.

Next, we aim to confirm the effects of three Ca^2+^ modulators (2-APB, TG and BAPTA-AM) on the [Ca^2+^]_c_ and [Ca^2+^]_ER_ in Pb-exposed rPT cells. 2-APB is a specific inhibitor of inositol 1, 4, 5-trisphosphate receptor (IP_3_R) that functions to release Ca^2+^ from ER stores [16]. TG raises cytosolic calcium concentration by inhibiting the ER-Ca^2+^-ATPase [17], while BAPTA-AM is an intracellular Ca^2+^ chelator to attenuate the elevation of [Ca^2+^]_c_ [18]. Data in Figure 2A showed that treatment with 2-APB or BAPTA-AM significantly suppressed Pb-induced [Ca^2+^]_c_ elevation, respectively; 2-APB or BAPTA-AM treatment alone has no obvious effect on [Ca^2+^]_c_ in rPT cells. However, treatment with TG further aggravated Pb-mediated cytosolic Ca^2+^ overload and TG treatment only caused significant cytosolic Ca^2+^ elevation in rPT cells. As shown in Figure 2B, treatment with 2-APB significantly inhibited Pb-induced [Ca^2+^]_ER_ depletion while TG treatment further aggravated Pb-mediated ER Ca^2+^ release. Treatment with BAPTA-AM has no effect on Pb-induced [Ca^2+^]_ER_. Neither 2-APB nor BAPTA-AM treatment only has no obvious effect on [Ca^2+^]_ER_ in rPT cells, and TG treatment alone caused significant ER Ca^2+^ release in rPT cells. Data in Figure 2 give us a solid evidence that these three chemicals can efficiently regulate Pb-induced [Ca^2+^]_c_ elevation and [Ca^2+^]_ER_ depletion.

### Pb-mediated ER stress is Ca^2+^-dependent

**Figure 3:**
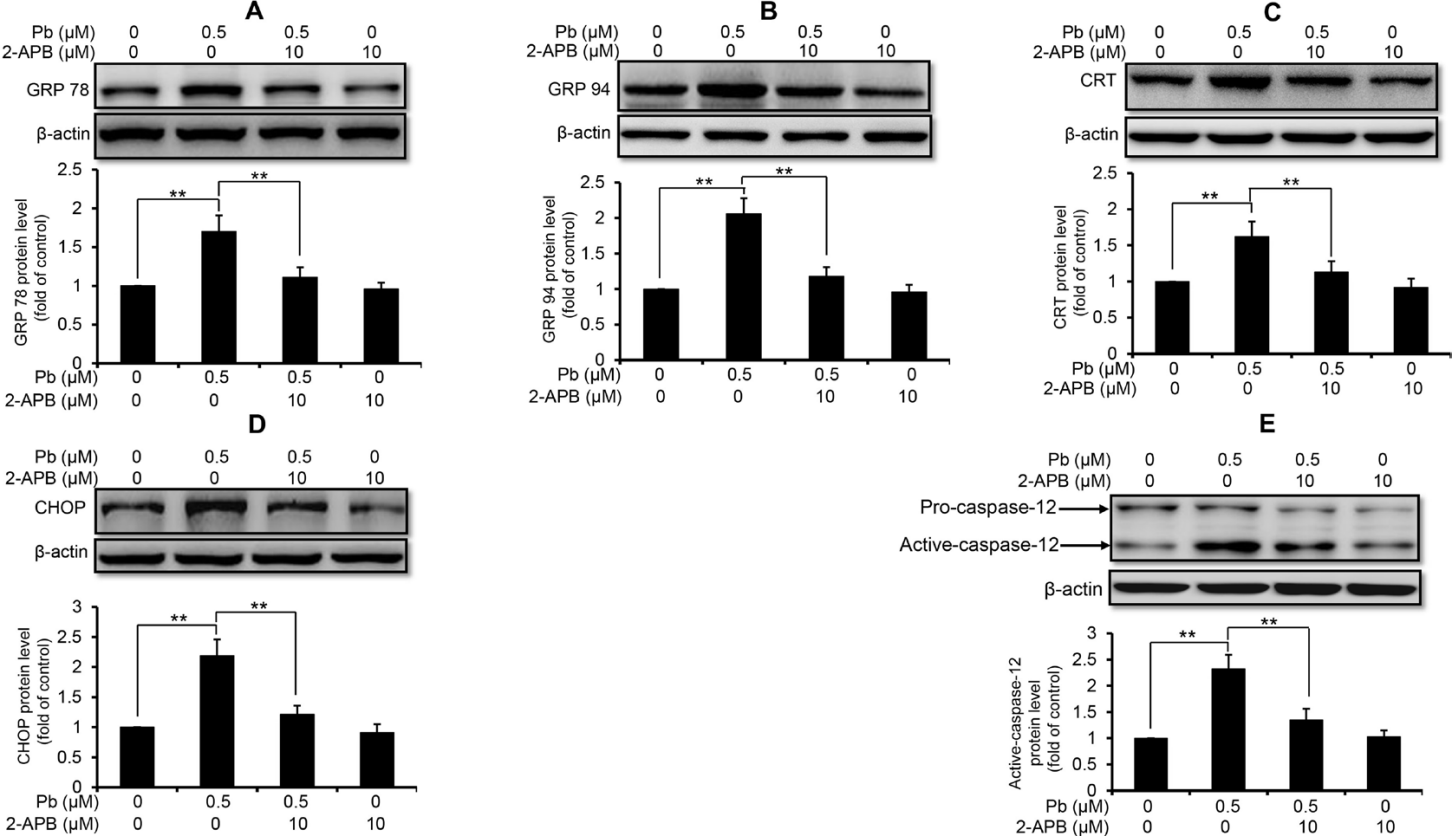
2-APB inhibited the protein expression of ER stress markers in Pb-exposed cells Cells were co-treated with 10 µM 2-APB and/or 0.5 µM Pb for 12 h, then harvested to measure the protein levels of GRP78 (**A**), GRP94 (**B**), CRT (**C**), CHOP (**D**) and caspase-12 (**E**). *Upper panel* representative western blot image; *lower panel* quantitative analysis (mean ± SEM, *n* = 4). ^**^P < 0.01.

**Figure 4:**
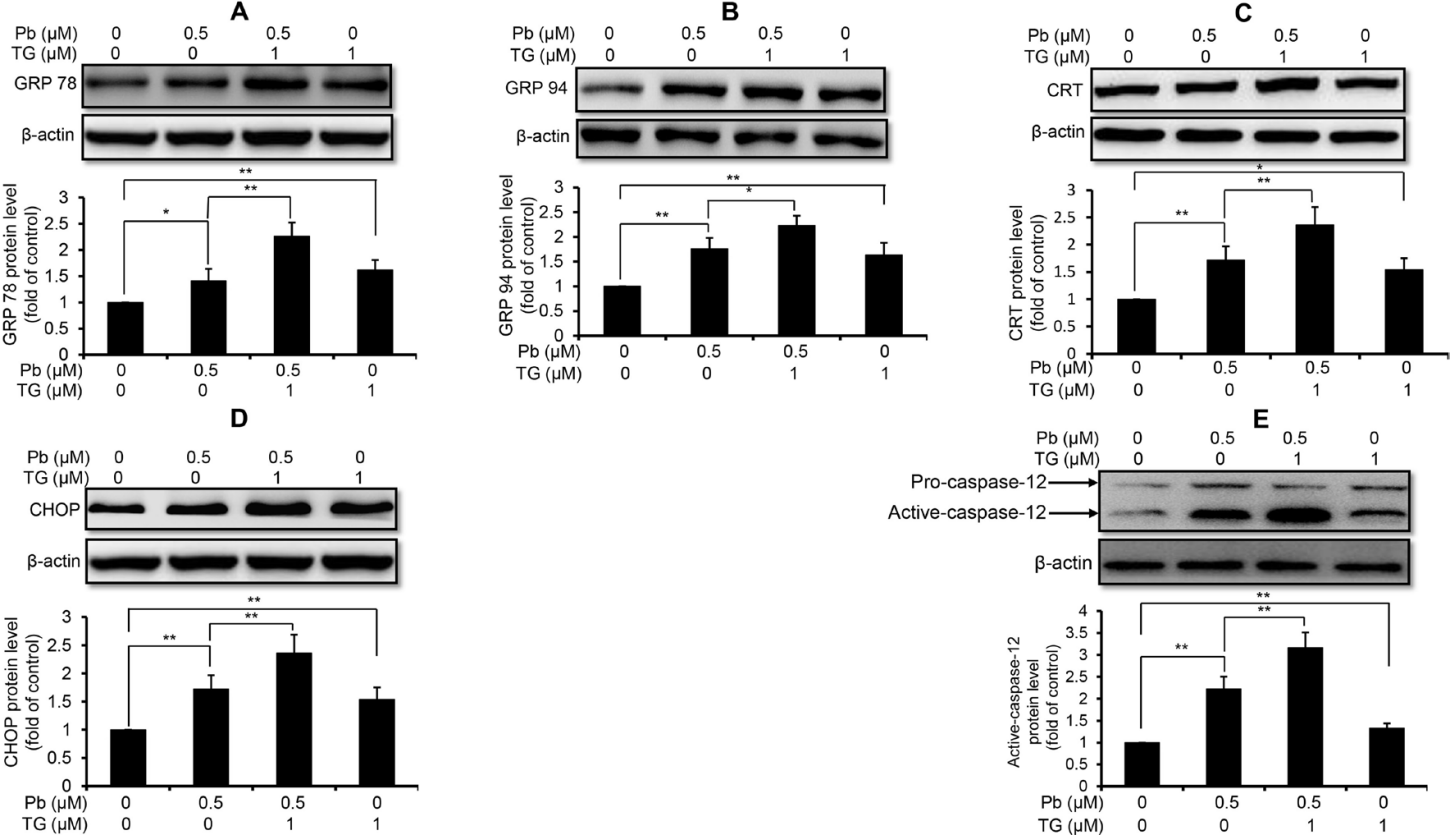
TG aggravated Pb-elevated protein levels of ER stress markers in rPT cells Cells were co-incubated with 1 µM TG and/or 0.5 µM Pb for 12 h to detect the protein levels of GRP78 (**A**), GRP94 (**B**), CRT (**C**), CHOP (**D**) and caspase-12 (**E**). *Upper panel* representative western blot image; *lower panel* quantitative analysis (mean ± SEM, *n* = 4). ^*^P < 0.05, ^**^P < 0.01.

**Figure 5:**
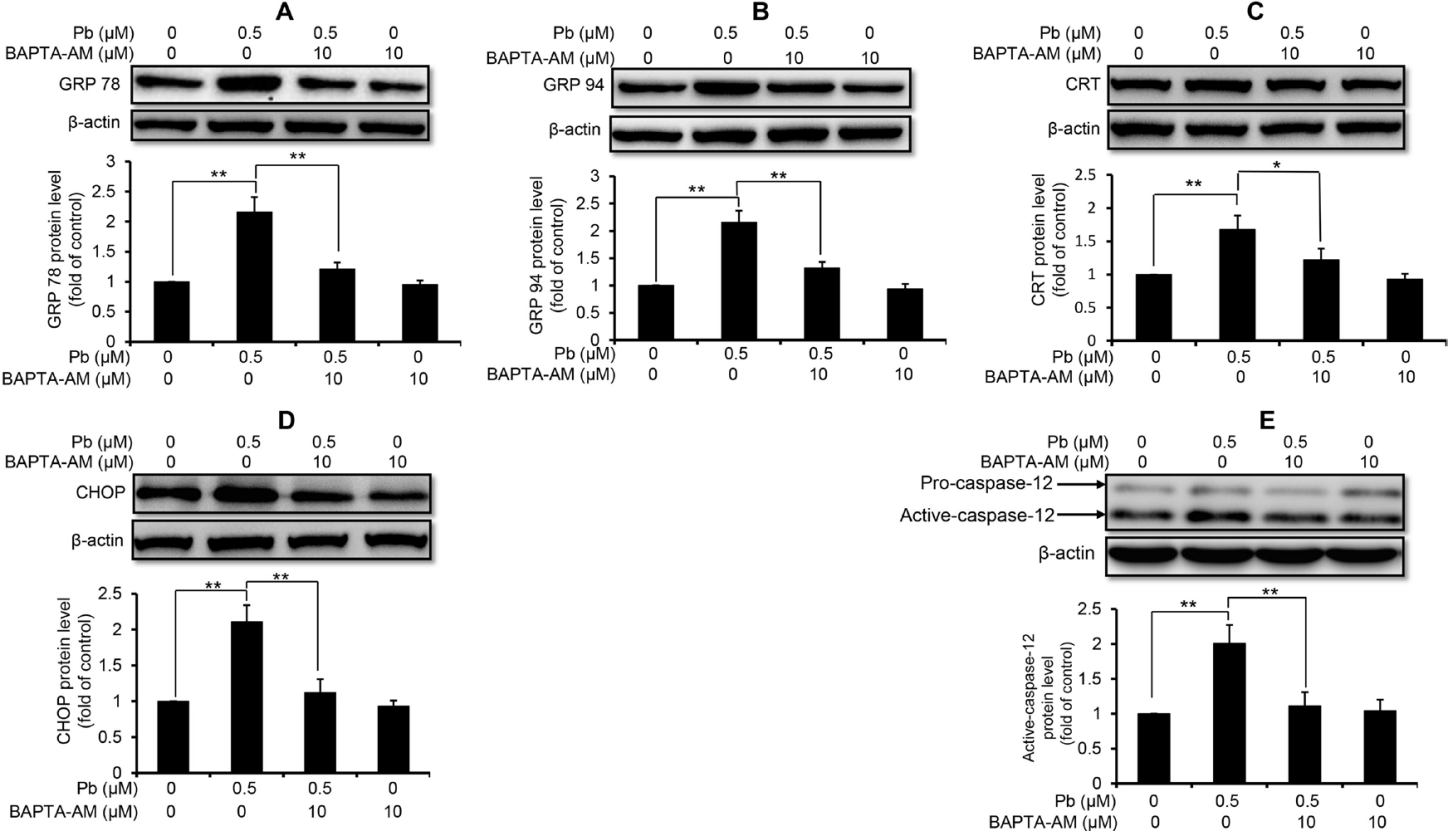
Effect of BAPTA-AM on Pb-induced protein levels of ER stress markers in rPT cells Cells were pretreated with 10 µM BAPTA-AM for 30 min, then exposed to 0.5 µM Pb for another 12 h to measure the protein levels of GRP78 (**A**), GRP94 (**B**), CRT (**C**), CHOP (**D**) and caspase-12 (**E**). *Upper panel* representative western blot image; *lower panel* quantitative analysis (mean ± SEM, *n* = 4). ^*^P < 0.05, ^**^P < 0.01.

Next, we used these three intracellular Ca^2+^ modulators to explore the role of Ca^2+^ signaling in Pb-induced ER stress in rPT cells. As shown in Figure 3, inhibition of ER Ca^2+^ release with 2-APB markedly diminished the protein levels of GRP78 (A), GRP94 (B), CRT (C), CHOP (D) and active caspaes-12 (E) in Pb-exposed rPT cells. In contrast, induction of ER Ca^2+^ release with TG significantly aggravated the protein levels of GRP78 (A), GRP94 (B), CRT (C), CHOP (D) and active caspaes-12 (E) in Pb-exposed rPT cells (Figure 4). Data in Figure 3 and Figure 4 clearly indicated that the ER Ca^2+^ release plays a crucial role in Pb-mediated ER stress in rPT cells. Additionally, data in Figure 5 showed that chelation of cytosolic Ca^2+^ with BAPTA-AM significantly suppressed the elevation of these five ER stress marker protein levels in Pb-exposed rPT cells. Based on the changes of [Ca^2+^]_c_ regulated by three Ca^2+^ modulators, these data sufficiently demonstrated the regulatory effect of elevated cytosolic Ca^2+^ on Pb-induced ER stress in rPT cells. Collectively, Pb-induced ER stress in rPT cells is Ca^2+^ dependent.

### CaMKII activation is involved in Pb-exposed rPT cells

**Figure 6:**
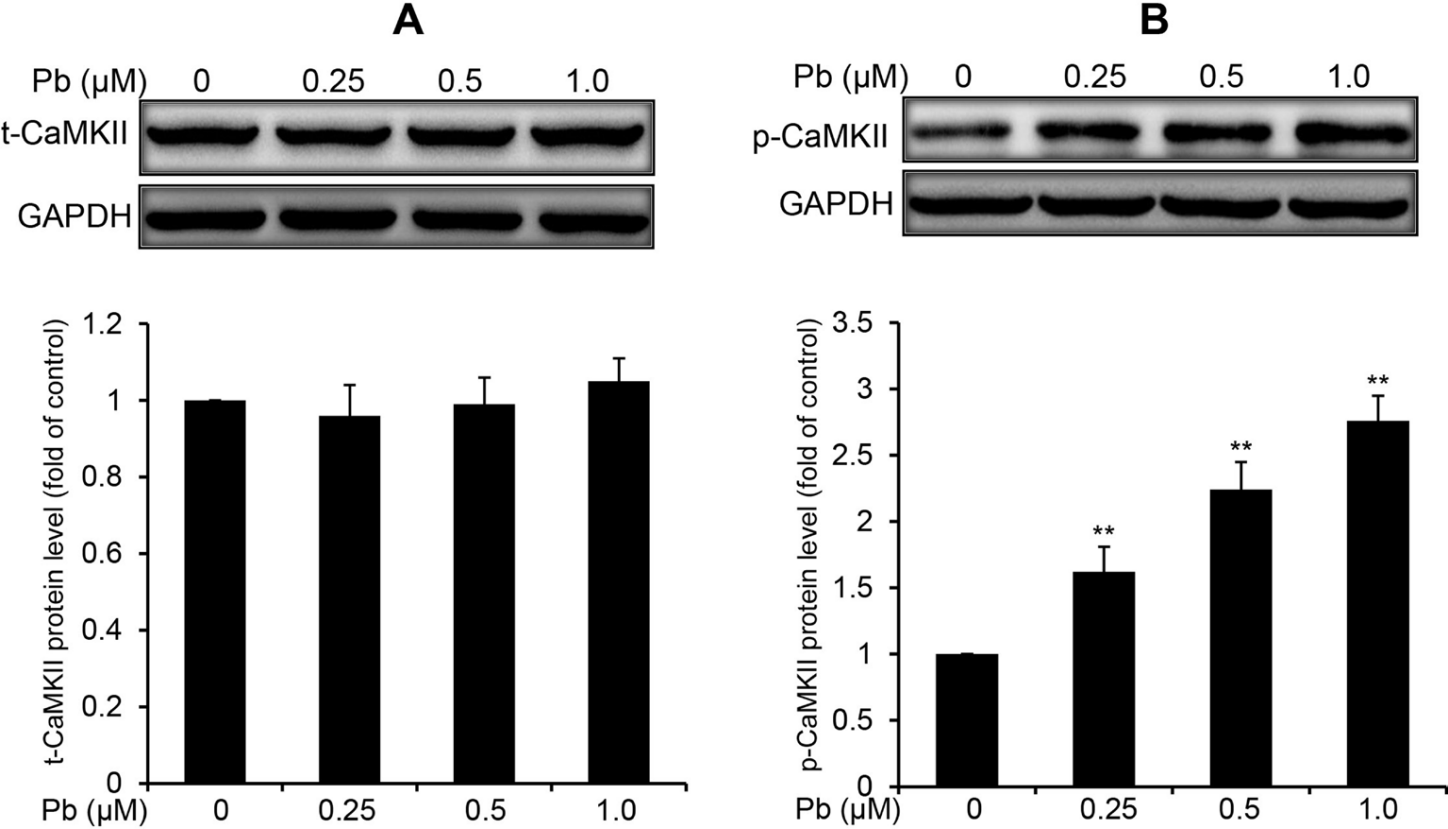
Pb elevated the phosphorylation of CaMKII in rPT cells Cells were treated with Pb(NO_3_)_2_ (0.25, 0.5 and 1 µM) for 12 h, then collected to analyze the protein levels of t-CaMKII (**A**) and p-CaMKII (**B**). *Upper panel* representative western blot image; *lower panel* quantitative analysis (mean ± SEM, *n* = 4). ^**^P < 0.01.

**Figure 7:**
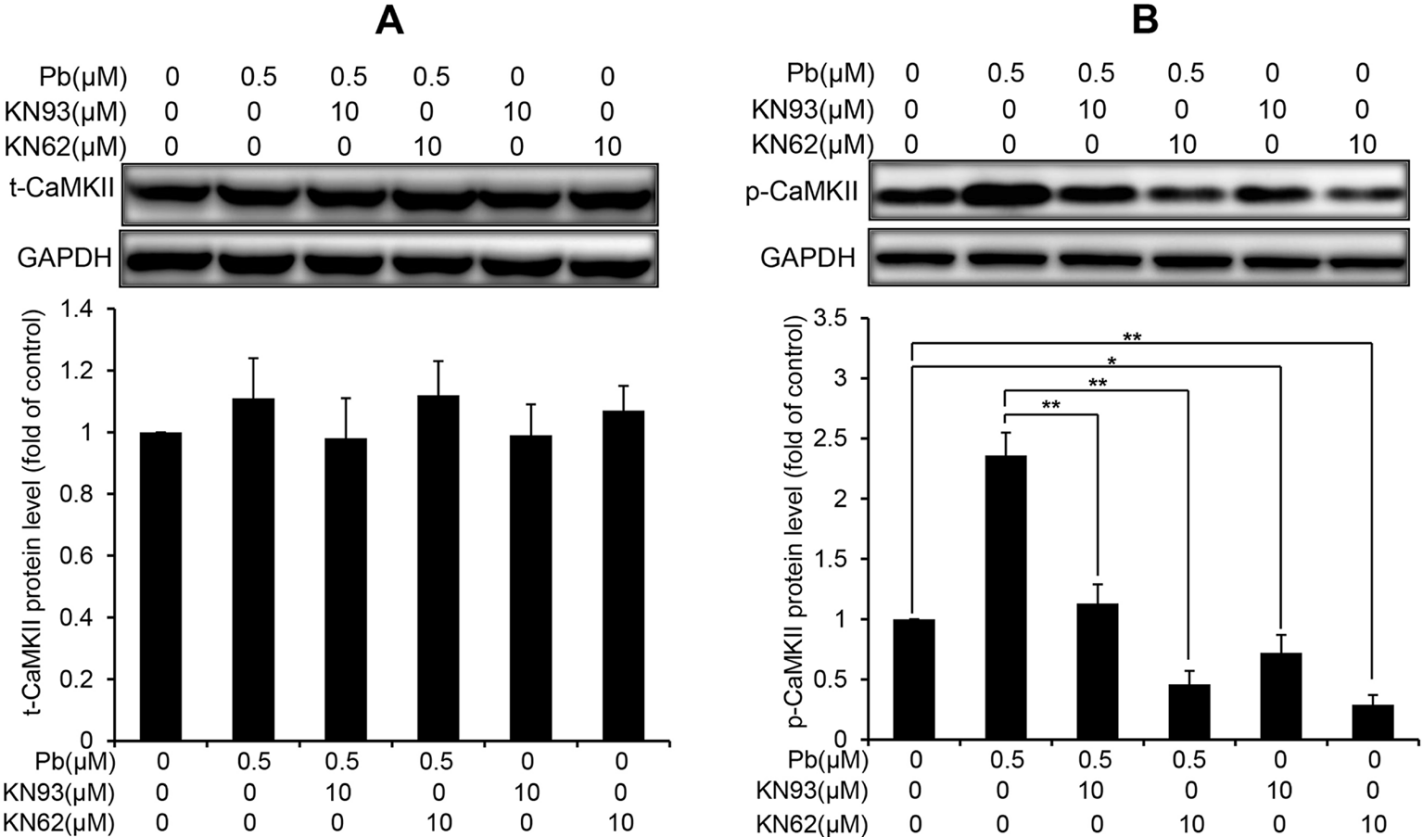
Effects of two CaMKII inhibitors (KN93, KN62) on the protein levels of t-CaMKII and p-CaMKII in Pb-exposed cells Cells were pre-incubated with 10 µM KN93 or 10 µM KN62 for 2 h, then exposed to 0.5 µM Pb for another 12 h to measure the protein levels of t-CaMKII (**A**) and p-CaMKII (**B**), respectively. *Upper panel* representative western blot image; *lower panel* quantitative analysis (mean ± SEM, *n* = 4). ^*^P < 0.05, ^**^P < 0.01.

CaMKII is an important mediator of Ca^2+^ signaling in cells, and canonically activated by the elevation of intracellular Ca^2+^ [19]. Thus, we examined the CaMKII protein expression and phospho-activation in Pb-treated rPT cells. Western blot analysis showed that the total CaMKII protein levels were not affected by Pb treatment compared to control cells (Figure 6A). In contrast, CaMKII kinase activation, as indicated by CaMKII phosphorylation (p-CaMKII expression), was revealed in Pb-exposed rPT cells (Figure 6B). Simultaneously, two classical CaMKII inhibitors KN62 and KN93 were applied to validate the CaMKII activation in Pb-exposed cells (Figure 7). As expected, pre-treatment with KN62 or KN93 had no effect on total CaMKII expression in Pb-exposed rPT cells. However, Pb-elevated p-CaMKII level was blocked by pre-treatment with KN62 or KN93, respectively. Given these results, CaMKII activation is involved in Pb-exposed rPT cells.

### CaMKII inhibition blocks Pb-induced ER stress and apoptosis in rPT cells

**Figure 8:**
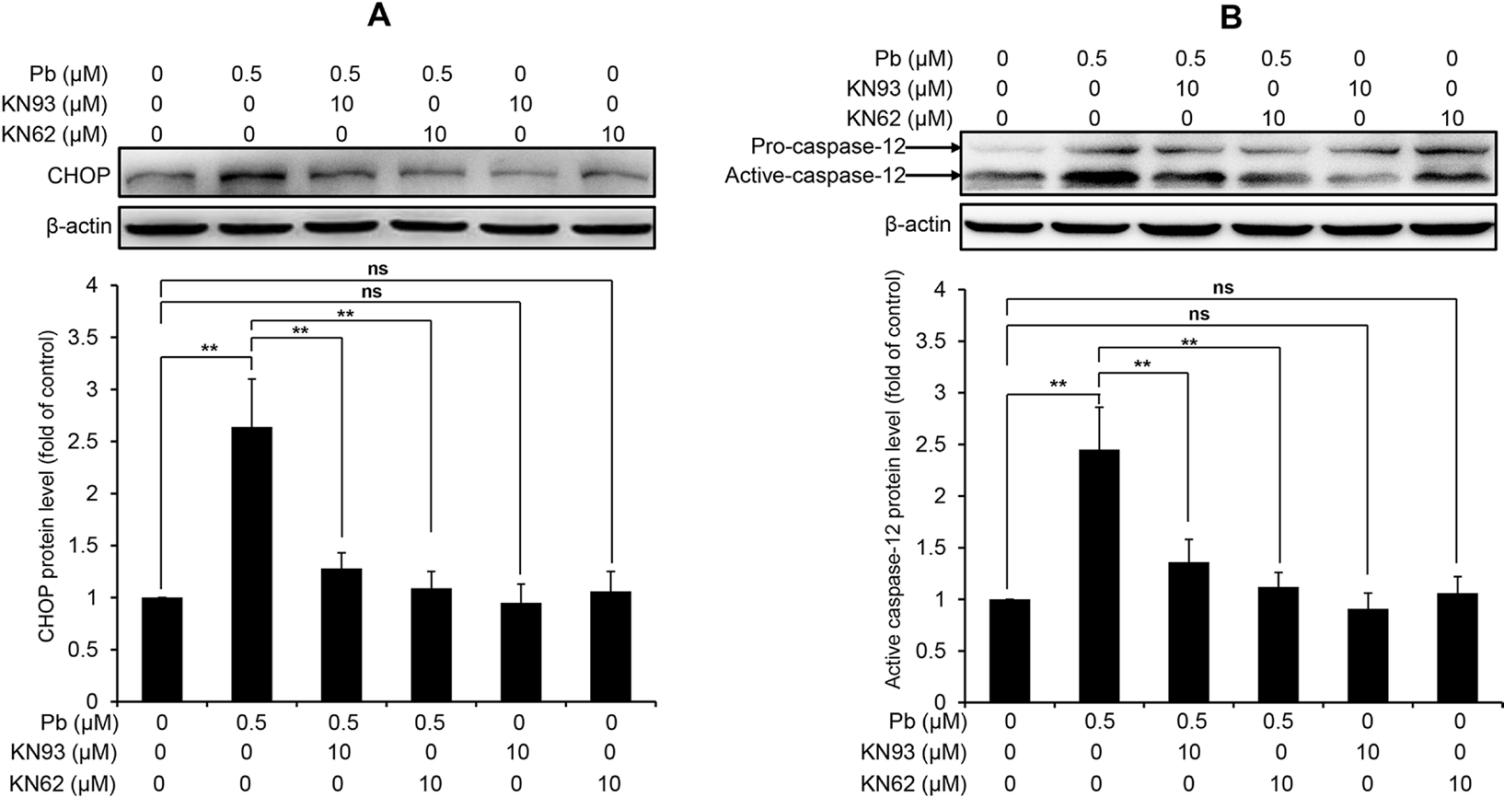
CaMKII inhibition blocks the activation of CHOP and caspase-12 in Pb-exposed rPT cells Cells were pre-incubated with 10 µM KN93 or 10 µM KN62 for 2 h, then exposed to 0.5 µM Pb for another 12 h to measure the protein levels of CHOP (**A**) and caspase-12 (**B**), respectively. *Upper panel* representative western blot image; *lower panel* quantitative analysis (mean ± SEM, *n* = 4). *ns* not significant; ^**^P < 0.01.

**Figure 9:**
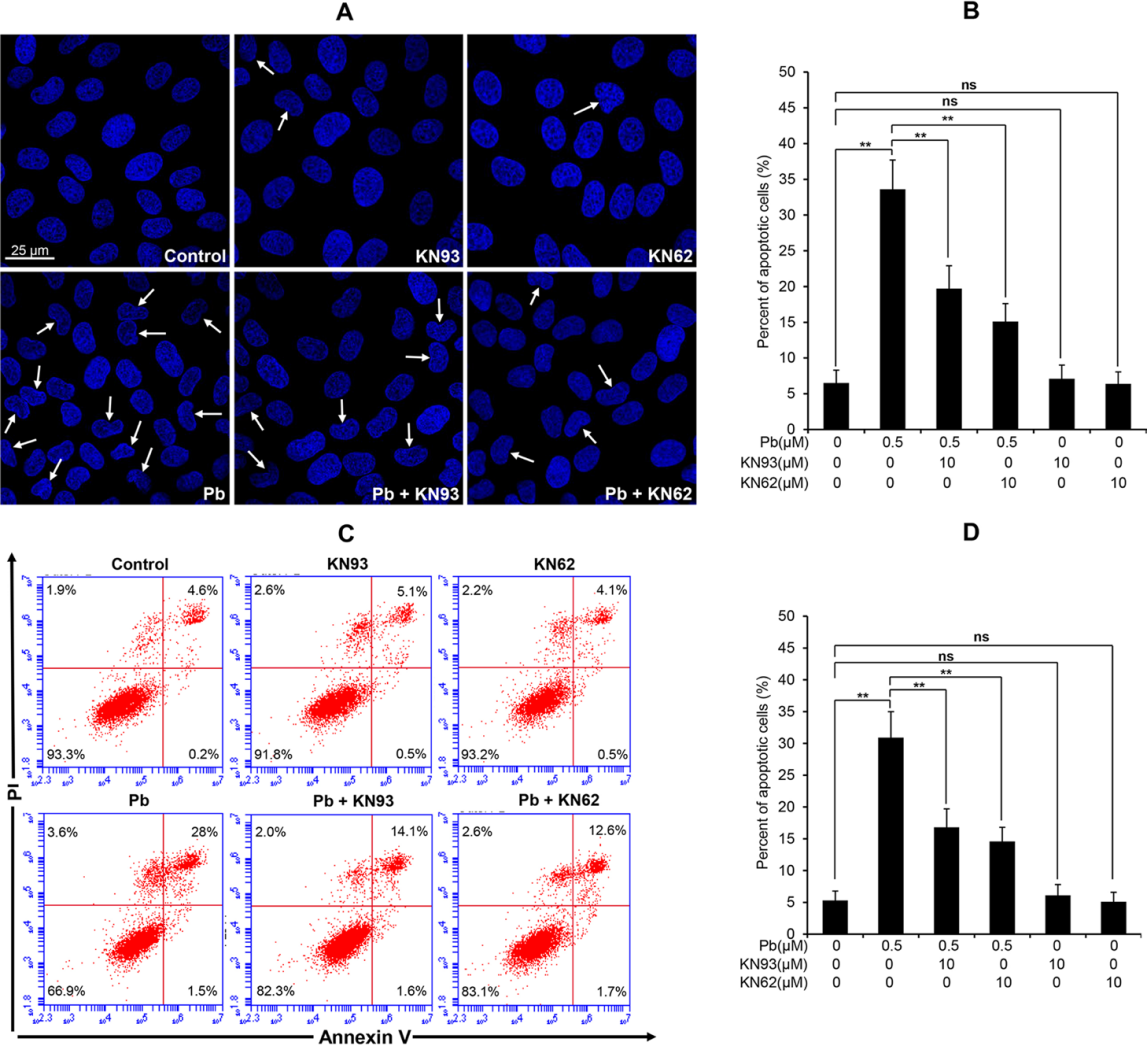
Effects of two CaMKII inhibitors (KN93, KN62) on Pb-induced apoptosis in rPT cells (**A**, **B**) Cells grown on coverslips were pre-incubated with 10 µM KN93 or 10 µM KN62 for 2 h, then exposed to 0.5 µM Pb for another 12 h to assess the apoptosis using Hoechst 33258 staining. Representative morphological changes of apoptosis are present in (A), and its statistical result of apoptotic rates (B) are expressed as mean ± SEM (*n* = 9). (**C**, **D**) Cells were pre-treated with 10 µM KN93 or 10 µM KN62 for 2 h, then exposed to 0.5 µM Pb for 12 h to assess the apoptosis using flow cytometry. Data in (D) are mean ± SEM of three separate experiments, and each one performed in triplicate (*n* = 9). *ns* not significant; ** *P <* 0.01.

CaMKII is a well-known mediator of apoptosis, while activated CaMKII can result in ER stress-induced apoptosis [15]. CHOP and active caspase-12 are two important mediators in ER stress-induced apoptosis. Firstly, we performed immunoblots to verify the effect of pharmacologic inhibition of CaMKII on the changes of two ER stress markers in Pb-exposed cells. Data in Figure 8 showed that Pb-elevated CHOP and active caspase-12 protein levels were significantly blunted with CaKMII inhibitor KN62 or KN93, respectively. To further assess whether CaMKII activation contributes to Pb-induced apoptosis, two CaMKII inhibitors (KN93 and KN62) were applied to prove this issue. As shown in Figure 9, Pb-induced apoptosis was significantly inhibited by specific CaMKII inhibitor KN93 or KN62, respectively. Neither KN93 nor KN62 treatment alone affected apoptosis in rPT cells. Collectively, it is reasonable to suggest that CaMKII activation promotes ER stress-induced apoptosis in Pb-exposed rPT cells.

### CaMKII controls intracellular Ca^2+^ level and ER Ca^2+^ release in Pb-exposed rPT cells

**Figure 10:**
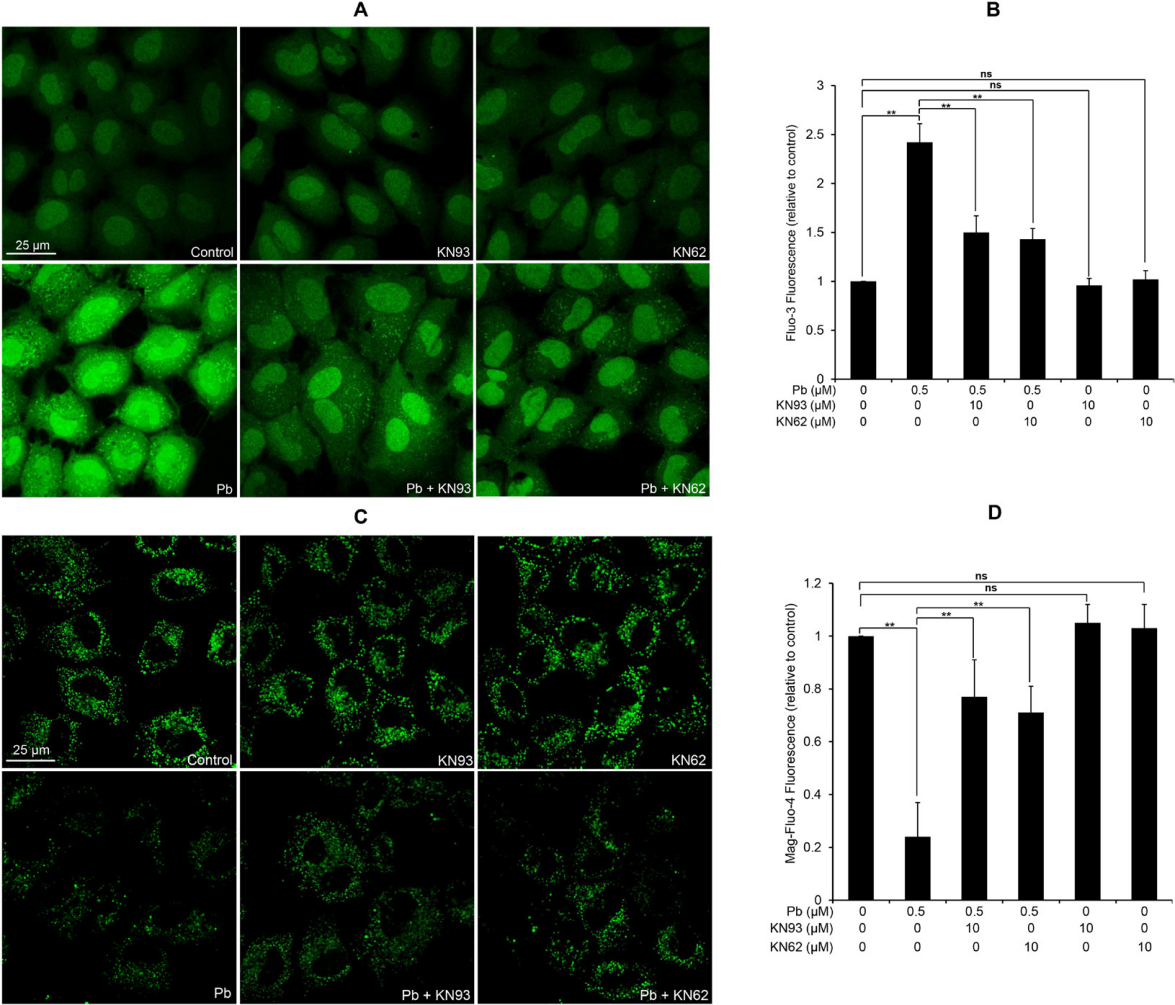
Modulation of [Ca^2+^]c and [Ca^2+^]ER by two CaMKII inhibitors in Pb-exposed rPT cells Cells were pre-treated with 10 µM KN93 or 10 µM KN62 for 2 h, then exposed to 0.5 µM Pb for another 12 h to measure the changes of [Ca^2+^]_c_ and [Ca^2+^]_ER_ using confocal microscopy, respectively. (**A**, **C**) Representative confocal images. (**B**, **D**) Quantification of fluorescence intensity from two Ca^2+^ indicators. The values of Fluo-3 fluorescence (B) and Mag-Fluo-4 fluorescence (D) are quantified in a relative way to its respective control group, whose value is set at “1”. Data represent mean ± SEM of three separate experiments and each one performed in duplicate (*n* = 6). *ns* not significant; ** *P <* 0.01.

Since activated CaMKII promotes apoptosis partly through regulation of ER Ca^2+^ uptake and CaMKII inhibition significantly reduced ER Ca^2+^ levels [20], we next assessed whether there is a link between CaMKII activation and subcellular Ca^2+^ redistribution in Pb-exposed rPT cells. As shown in Figure 10, 10 µM KN93 or 10 µM KN62 treatment alone has no obvious effect on the changes of [Ca^2+^]_c_ and [Ca^2+^]_ER_ in rPT cells, compared with the control group. Pb exposure resulted in a robust increase in [Ca^2+^]_c_ and decrease in [Ca^2+^]_ER_, while co-treatment with KN93 or KN62 significantly reversed the Pb-induced subcellular Ca^2+^ redistribution. These findings were confirmed in separate experiments by confocal microscopy with the fluorescent dye Fluo-3 and Mag-Fluo-4. Our data demonstrate that CaMKII activation is required for Pb-induced subcellular calcium redistribution (ER Ca^2+^ release and concomitant intracellular Ca^2+^ overload) in rPT cells.

## DISCUSSION

Lead is a well-known nephrotoxicant, of which proximal tubular epithelium is the main site of Pb-induced nephrotoxicity [21, 22]. It has been previously revealed that Pb induces apoptosis in rPT cells by triggering ER-Ca^2+^ release and cytosolic Ca^2+^ overload [9], but the detailed mechanism underlying Ca^2+^ dyshomeostasis-associated apoptosis remains to be elucidated. Herein, the present experiments provide evidences to demonstrate the effect of disturbed Ca^2+^ signaling on ER stress, and address the role of CaMKII in ER stress, subcellular Ca^2+^ redistribution and apoptosis in Pb-exposed rPT cells.

ER performs crucial cellular functions and acts as the largest dynamic intracellular Ca^2+^-storage organelle. ER dysfunction can lead to an imbalance between protein-folding capacity and protein-folding load as well as Ca^2+^ dyshomeostasis, which in turn leads to ER stress [23]. Adaptive ER stress is initially aimed at ensuring cell survival by activating the transcription of ER chaperones, such as GRP78, GRP94 and CRT [23]. If the ER stress is too severe or prolonged to repair, the stressed cell will initiate apoptotic cell death through several signaling cascades, such as caspase-12 and CHOP [24]. Caspase-12, predominantly localized on the cytoplasmic side of the ER, is a regulator specific to ER stress-induced apoptosis [25]. Activation of caspase-12 (active caspase-12) from procaspase-12 is specifically induced by excessive or prolonged ER stress, which initiates the apoptotic death cascades [26]. CHOP is a transcription factor to induce several proapoptotic factors, which has been identified as one of the most important mediators in ER stress-induced apoptosis [27]. Noteworthy, ER stress has been described as a contributing factor to tubular damage in kidney diseases because tubular cells are particularly sensitive to ER stress [28]. However, the role of ER stress in Pb-induced nephrotoxicity has not yet been reported in previous studies. In this study, elevated protein levels of GRP78, GRP94 and CRT as well as activation of CHOP and caspase-12 were revealed in Pb-exposed rPT cells (Figure 1), indicating that ER stress and ER stress-mediated apoptosis are involved in Pb-induced nephrotoxicity.

It is important to note that ER-Ca^2+^ homeostasis is crucial to ensure the proper protein-folding, while ER Ca^2+^ imbalance can greatly impact the folding capacity and induce ER stress-mediated apoptosis [29–31]. Our previous studies have demonstrated that Pb causes Ca^2+^ dysregulation in rPT cells [9], which arises our interest to investigate the correlation between ER stress and Ca^2+^ dyshomeostasis in Pb-exposed rPT cells. Hereby, 2-APB (the blocker of ER Ca^2+^ release channel-IP_3_R), TG (an ER Ca^2+^ pump inhibitor) and BAPTA-AM (cytosolic Ca^2+^ chelator) was chosen to modulate the changes of [Ca^2+^]_c_ and [Ca^2+^]_ER_ in this study, respectively. As expected, three modulators of Ca^2+^ signaling can potently regulate the oscillations of [Ca^2+^]_c_ and [Ca^2+^]_ER_ in Pb-treated rPT cells (Figure 2). Next, we assessed the effects of three Ca^2+^ modulators on protein levels of five ER stress markers in Pb-treated rPT cells. Data from this study (Figures 3–5) give us a solid conclusion that subcellular calcium redistribution acts as an important pathological mediator of ER stress in Pb-exposed rPT cells, i.e., Pb-induced ER stress is Ca^2+^-dependent.

Calcium requires a variety of effectors to sustain signaling events. CaMKII has emerged as a key effector of Ca^2+^ signaling, which is involved in the regulation of Ca^2+^ mobilization [32, 33]. CaMKII is a multifunctional serine/threonine protein kinase present in many tissues, which activates several signaling cascades involved in cell survival or cell death [33]. Emerging evidence suggests CaMKII evoked apoptotic cell death, is one of the key underlying mechanisms for the detrimental effect of sustained CaMKII activation [34]. While CaMKII function is well defined in cardiac myocytes and neurons [30], the role of CaMKII in Pb-induced nephrotoxicity has not been reported up to date. Data in Figure 6 verified the activation of CaMKII in Pb-exposed rPT cells, which gave us a hint that activated CaMKII may be related to Pb-induced apoptosis in rPT cells.

Moreover, CaMKII is emerging as a central signaling effector involved in the amplification of oxidative stress initiated by the CHOP-mediated maladaptive ER stress response pathway to promote apoptosis [15]. Also, activated CaMKII can result in ER stress-induced apoptosis through activation of caspase-12 pathway [30]. Next, we used two CaMKII inhibitors, i.e., KN93 and KN62, which can potently block CaMKII activation (Figure 7), to investigate the role of CaMKII activation in Pb-induced ER stress and associated apoptosis in rPT cells. Firstly, western-blot analysis results (Figure 8) showed that Pb-induced activation of CHOP and caspase-12 was effectively blocked by KN93 or KN62, verifying the promoting effect of CaMKII activation in Pb-induced ER stress in rPT cells. Secondly, Pb-induced apoptosis (Figure 9), assessed by two methods, can be significantly inhibited by the co-treatment of KN93 or KN62, demonstrating the regulatory role of CaMKII activation in Pb-induced apoptosis. Collectively, it can be concluded that CaMKII is an important mediator in ER stress and associated apoptosis in Pb-exposed rPT cells.

Additionally, CaMKII activation is an upstream signaling event for promoting ER-Ca^2+^ release [14]. Given the regulatory role of CaMKII activation in ER stress-induced apoptosis, we hypothesized that there is a certain relationship between CaMKII activation and subcellular calcium redistribution in Pb-exposed rPT cells. As expected, Pb-induced subcellular calcium redistribution, i.e., increase in [Ca^2+^]_c_ and concomitant decrease in [Ca^2+^]_ER_, was effectively blocked by CaMKII antagonist KN93 or KN62 (Figure 10), indicating the controller role of CaMKII in this process. However, the detailed mechanism underlying CaMKII regulates intracellular Ca^2+^ dynamics in Pb-exposed rPT cells remains to be further clarified.

In summary, Pb induced ER stress in rPT cells, which is dependent on Ca^2+^ signaling. Our findings demonstrated an essential role of CaMKII in Pb-induced nephrotoxicity. Activated CaMKII acts as an important intermediate in Pb-induced ER stress and associated apoptosis in rPT cells, mainly through promoting subcellular calcium redistribution.

## MATERIALS AND METHODS

### Chemicals and antibodies

All chemicals were of the highest grade purity available. Lead nitrate, collagenase IV, trypsin, Thapsigargin (TG), DMEM-F12 (1:1), Hoechst 33258 solution and all other chemicals were purchased from Sigma-Aldrich. A phosphatase inhibitor (04906845001) was obtained from Roche Applied Science. Pluronic F-127 and Fluo-3-AM were obtained from Dojindo Laboratories (Tokyo, Japan). Mag-Fluo-4-AM and N, N, N′, N′-tetrakis-(2-pyridylmethyl) ethylenediamine (TPEN) were purchased from Molecular Probes (Eugene, OR, USA). KN62 and KN93 were purchased from Selleck (Houston, Texas, USA). 2-Aminoethoxydiphenyl borate (2-APB) and 1, 2-Bis (2-aminophenoxy) ethane-N, N, N’, N’-tetraacetic acid acetoxymethyl ester (BAPTA-AM) were from Tocris Bioscience (Bristol, UK). Annexin V-FITC apoptosis detection kit was from Pharmingen (Becton Dickinson Company, USA). BCA protein assay kit and enhanced chemiluminescence (ECL) kit were obtained from Thermo Fisher Scientific Pierce. The following primary antibodies were used: GRP78 (cat No. 3183), GRP94 (cat No. 2104), Calreticulin (CRT, cat No. 12238), phospho-CaMKII (p-CaMKII, cat No. 12716), β-actin (cat No. 3700) and GAPDH (cat No. 5174) were purchased from Cell Signaling Technology. CHOP (cat No. ab11419), Caspase-12 (cat No. ab62484) and total-CaMKII (t-CaMKII, cat No. ab52476) were obtained from Abcam. Secondary antibodies were conjugated to horseradish peroxidase (Jackson Immuno Research, cat No. 705-505-303 and cat No. 111-006-062).

### Cell culture and treatment

All procedures followed the ethics guidelines and were approved by the Animal Care and Use Committee of Shandong Agricultural University (SDAUA-2017-003). Isolation, identification and culture of Sprague-Dawley rat proximal tubular (rPT) cells were as previously described [35]. BAPTA-AM, TG, 2-APB, KN93 and KN62 were dissolved in DMSO to make the stock solutions, filtered and stored at −20°C, then diluted to work solution prior to use. The final concentration of DMSO was less than 0.1% and 0.1% DMSO has no effect on Ca^2+^ signaling and cell viability [36]. Meanwhile, 2-APB, BAPTA-AM, KN93 and KN62 have no significant toxic effects on cells, as confirmed in this study (data not shown). Based on the doses of Pb in our previous study [37], cell cultures were incubated in the presence of 0, 0.25, 0.5, and 1 µM Pb. Primarily, events of Pb exposure over a 12-h period were chosen to investigate the Pb-induced cytotoxicity.

### Flow cytometric measurement of cytosolic Ca^2+^ concentration ([Ca^2+^]_c_)

Three chemicals, i.e., BAPTA-AM (cytosolic Ca^2+^ chelator), 2-APB {an inhibitor of inositol 1, 4, 5-trisphosphate receptor (IP_3_R)} and TG (a potent ER Ca^2+^ pump inhibitor) were chosen to investigate the changes of [Ca^2+^]_c_ in this study. rPT cells were pretreated with 10 µM BAPTA-AM for 30 min, followed by incubation with 0.5 µM Pb for another 12 h. Also, cells were co-incubated with 10 µM 2-APB and 2.5 µM Cd or 1 µM TG and 2.5 µM Cd for 12 h. After the treatment, harvested cells were incubated with 0.5 mM of TPEN at 37°C for 10 min, loaded with 1 µM Fluo-3-AM (containing 0.02% Pluronic F-127) for 30 min in dark at 37°C, and then washed with D-Hank’s solution. Intracellular Ca^2+^ levels were represented with fluorescent intensity (FL-1, 530 nm) of 10,000 cells on flow cytometer. This assay was replicated six different times using different batches of cells.

### Flow cytometric analysis of endoplasmic reticulum Ca^2+^ levels ([Ca^2+^]_ER_)

It has been verified that Mag-Fluo-4-AM was selectively labelled on ER, making its specificity for measuring [Ca^2+^]_ER_ [9]. After corresponding treatment, cells were incubated with 2 µM Mag-Fluo-4-AM and 0.02% (w/v) Pluronic F-127 for 30 min at 37°C in dark. Pb was removed by treatment of TPEN as described above. 488-nm laser was used to excite Mag-Fluo-4 fluorescence and [Ca^2+^]_ER_ was calculated by the fluorescence intensity (FL-1, 530 nm) of 10,000 cells on flow cytometer.

### Western blot analysis

Cells subjected to desired treatments were lysed in ice-cold RIPA buffer supplemented with protease inhibitor (Merck Millipore) and phosphatase inhibitor (Roche) to prepare the total cell lysates. After protein quantification with BCA method, samples were subjected to SDS-PAGE gels and transferred to PVDF membranes. After blocking with 5% skim milk for 1 h at room temperature, membranes were incubated overnight at 4°C with the following primary antibodies: GRP78 (diluted 1:1000), GRP94 (diluted 1:1000), CRT (diluted 1:1000), CHOP (diluted 1:1000), Caspase-12 (diluted 1:1000), t-CaMKII (diluted 1:1250), p-CaMKII (diluted 1:1000), β-actin (diluted 1:1000) and GAPDH (diluted 1:1000). After several washes with TBST, the membranes were incubated with appropriate secondary antibodies (1:5000 dilution) for 50 min at room temperature. Finally, each protein was detected on a Chemidoc XRS (Bio-Rad, Marnes-La-Coquette, France) by using the ECL kit. Proteins levels were determined by computer-assisted densitometric analysis (Densitometer, GS-800, BioRad Quantity One). The density of each band was normalized to its respective loading control (β-actin or GAPDH). Data obtained were expressed as the ratio of intensity of the protein in chemical-treated cells to that of the corresponding protein in control cells. Each test was performed in four experiments with different batches of cells.

### Assessment of apoptosis by morphological changes and flow cytometry

Cells were pre-incubated with 10 µM KN62 or 10 µM KN93 for 2 h, then treated with 0.5 µM Pb for another 12 h to assess its effect on apoptosis, respectively. Apoptosis is characterized morphologically by condensation and fragmentation of nuclei. Thus, Hoechst 33258 staining was firstly applied to assess the morphological changes of treated cells, and 200 cells were randomly selected to count those apoptotic cells within every batch of experiment, each one performed in triplicate. Another concern is the quantitative analysis of apoptosis by flow cytometry. Both of these two methods have been extensively described in our previous study [37].

### Analysis of the changes of [Ca^2+^]_c_ and [Ca^2+^]_ER_ by confocal microscopy

Two CaMKII inhibitors, i.e., KN62 and KN93, were applied to assess its effect on the changes of [Ca^2+^]_c_ and [Ca^2+^]_ER_ in Pb-exposed rPT cells, respectively. Cells grown on coverslips were pre-incubated with 10 µM KN62 or 10 µM KN93 for 2 h, then exposed to 0.5 µM Pb for another 12 h. After incubated with 0.5 mM of TPEN (Pb chelator) at 37°C for 10 min, one part of slides were loaded with 2.5 µM Fluo-3-AM (containing 0.02% Pluronic F-127) in dark at 37°C for 30 min, and the other part of slides were incubated with 2.5 µM Mag-Fluo-4-AM (containing 0.02% Pluronic F-127) in dark at 37°C for 30 min. After washing with D-Hank’s solution, cells labeled with Fluo-3-AM or Mag-Fluo-4-AM were visualized by the confocal microscope (TCS SPE, Leica, Germany) to assess the changes of [Ca^2+^]_c_ and [Ca^2+^]_ER_, respectively. Representative confocal images were captured. [Ca^2+^]_c_ and [Ca^2+^]_ER_ were quantified using the ImageJ software under identical threshold conditions.

### Data presentation

Experiments were performed at least three times with similar results. Data are presented as the mean ± SEM of the indicated number of replicates. Statistical comparisons were made using one-way analysis of variance (ANOVA) (Scheffe’s *F* test) after ascertaining the homogeneity of variance between the treatments, and *P* < 0.05 was regarded as significant.

## Abbreviations

2-APB: 2-Aminoethoxydiphenyl borate
BAPTA-AM: 1, 2-Bis (2-aminophenoxy) ethane-N, N, N’, N’-tetraacetic acid acetoxymethyl ester
BCA: bicinchonininc acid
CaMKII: Calcium/calmodulin-dependent protein kinase II
CHOP: CCAAT/enhancer-binding protein homologous protein
CRT: Calreticulin
[Ca^2+^]_c_: free Ca^2+^ concentration in the cytosol
[Ca^2+^]_ER_: free Ca^2+^ concentration in the ER
ECL: enhanced chemiluminescence
ER: endoplasmic reticulum
GRP78: glucose-regulated protein 78
GRP94: glucose-regulated protein 94
IP_3_: inositol 1, 4, 5-trisphosphate
IP_3_R: inositol 1, 4, 5-trisphosphate (IP_3_) receptor
TPEN: N, N, N′, N′-tetrakis-(2-pyridylmethyl) ethylenediamine
TG: Thapsigargin
rPT: rat proximal tubular
PI: propidium iodide
PBS: phosphate buffered saline
